# Fourier Analysis of Keratometric Data in Epithelium Removal versus Epithelial Disruption Corneal Cross-linking

**DOI:** 10.18502/jovr.v15i1.5934

**Published:** 2020-02-02

**Authors:** Shahram Bamdad, Seyed Mohammad Salar Zaheryani, Sahar Mohaghegh, Mohammad Shirvani

**Affiliations:** ^1^Poostchi Ophthalmology Research Center, Shiraz University of Medical Sciences, Shiraz, Iran; ^2^School of Rehabilitation, Shahid Beheshti University of Medical Science, Tehran, Iran

**Keywords:** Corneal Cross Linking, Epithelium Disruption, Epithelium Removal, Fourier Analysis, Keratoconus, Randomized Controlled Trial, Transepithelial

## Abstract

**Purpose:**

To compare epithelium-removal and epithelium-disruption corneal crosslinking (CXL) methods in Fourier analysis of keratometric data and clinical outcomes.

**Methods:**

In this double masked randomized clinical trial, each eye of 34 patients with bilateral keratoconus was randomly allocated to either the epithelium-removal or epithelium-disruption CXL treatment groups. Ocular examination, refraction, uncorrected and best spectacle-corrected visual acuity (UCVA and BSCVA, respectively) measurements, and Pentacam imaging (keratometry, pachymetry, and Fourier analysis) were performed at baseline and at six-month follow-up period.

**Results:**

Patients' mean age was 23.3 ± 3.6 years. The preoperative thickness of the thinnest point was 459.20 ± 37.40 µm and 455.80 ± 32.70 µm in the epithelium-removal and epithelial-disruption CXL groups, respectively (P > 0.05). The corresponding figures were 433.50 ± 33.50 µm and 451.90 ± 39.70 µm, respectively, six months after the treatment (P = 0.0001). Irregularity component of the fourier analysis was 0.030 ± 0.016 µm in the epithelium-removal group and 0.028 ± 0.011 µm in the epithelium-disruption group preoperatively (P > 0.05). This measurement was 0.031 ± 0.016 µm and 0.024 ± 0.009 µm, respectively at month 6 (P = 0.04). The epithelium-disruption CXL group had better results in terms of the thickness of the thinnest point and the irregularity component as compared to the epithelium-removal group. The two study groups were comparable in spherical equivalent, mean keratometry, UCVA, BSCVA, or other Fourier analysis components (spherical R min, spherical eccentricity, central, peripheral regular astigmatism, and maximum decentration) (P > 0.05).

**Conclusion:**

This study shows that epithelium-disruption CXL is superior to epithelium-removal CXL regarding the short-term changes in pachymetry and corneal irregularity. Other evaluated parameters were comparable between the two techniques.

##  INTRODUCTION

Keratoconus is a non-inflammatory bilateral progressive corneal ectasia characterized by corneal stromal thinning, protrusion, and irregular astigmatism that results in light scattering. It usually affects teenagers and young adults and causes decreased visual acuity.^[1]^ Keratoconus has a mental and economic burden on patients and drains the healthcare system budget.^[2,3]^ Its prevalence was reported to be 1 in 2,000 patients globally and 0.02% in the Tehran Eye Study.^[1,4]^

Wollensak et al introduced corneal collagen cross-linking (CXL)^[5]^ to halt the progression of keratoconus.^[6,7]^ Since then, CXL treatment has been used widely in clinics across the world. Its promising effects are also supported by a meta-analysis study.^[6]^ In the conventional CXL method, the epithelium of the central cornea (7–9 mm) is removed, and riboflavin solution is applied. Then, ultra-violet A is used to enhance crosslinking between adjacent corneal collagen fibers.^[5]^ Recently, some modifications were made to reduce the manipulation of the corneal epithelium, hence decreasing the initial pain, visual discomfort, and stromal haziness.^[8]^ One suggested procedure is trans-epithelial CXL in which hypo-osmolar riboflavin is applied to the cornea through an intact epithelium. The efficacy of this modification, however, was reported to be less than that of the conventional method in some studies.^[9,10]^ Therefore, partial epithelium-removal CXL was developed, which uses vertical and horizontal strips of de-epithelization using a custom-designed surgical instrument (Daya epithelium disruptor). The safety, efficacy, and better tolerability of this method have been reported in recent publications.^[11,12,13,14]^

Fourier analysis is a mathematical method that can convert periodic continuous data into unique expressions. Fourier analysis transforms the extracted keratometric data using a Pentacam Scheimpflug camera into spherical R min, spherical eccentricity, maximum decentration, central and peripheral astigmatism, and irregularity components. Fourier analysis can measure the 3-dimensional shape of the cornea, producing specific numbers that provides more comprehensive evaluation of cornea as compared to mean keratometric values. The repeatability and validity of Fourier analysis based on data from a Pentacam device were shown in a previous study by Sideroudi et al.^[15]^ This analysis provides a sensitive index for differentiating corneal ectasia and can be used for the prompt diagnosis of keratoconus.^[16]^ It has been shown that Fourier spherical and irregularity components change over a year in progressive keratoconus, but no significant changes occur in the other two components.^[17]^ The aim of this randomized clinical trial (RCT) study was to evaluate the whole shape of the cornea using Fourier analysis of keratometric data obtained from a PentacamⓇ HR (Oculus, Lynwood, WA, USA) and compare the results between epithelium-removal versus epithelium-disruption CXL techniques after six months of follow-up.

##  METHODS

##  Study Design

This was a double-masked RCT study that enrolled 34 bilateral keratoconus patients who had each eye examined and operated on at Khalili Hospital. Stratified randomization was performed for the eyes and methods; for each patient, one eye underwent epithelium-removal CXL and the other eye underwent epithelium-disruption CXL in two consecutive sessions with at least two weeks' interval. The study protocol was in accordance with the tenets of The Declaration of Helsinki and was approved by the local ethics committee of research at Shiraz University of Medical Sciences. Written informed consent was obtained from all participants. The RCT was registered at Iranian Registry of Clinical Trials (Number # IRCT2016112231028N1).

##  Patient Enrollment and Ocular Examination

The inclusion criteria were bilateral progressive keratoconus diagnosed according to the Rabinowitz criteria for patients aged 18–25 years. The progression of the disease was defined by an increase of at least 1 diopter (D) in mean keratometry (K mean) or a reduction of at least one line in the best-corrected visual acuity, within the last 12 months. The exclusion criteria were corneal thickness < 400 µm and K max > 61 D. In addition, pregnant and lactating women, and patients with history of previous ocular surgery, ocular herpetic infection, concurrent keratitis, severe corneal opacity, or ocular surface diseases such as dry eye, or autoimmune disease were excluded. The demographic and clinical characteristics of all patients were collected using data forms. Data including refraction, uncorrected and best spectacle-corrected visual acuity (UCVA & BSCVA, respectively) expressed in the logarithm of minimum angle of resolution scale (LogMAR), and data obtained using a PentacamⓇ HR device were compiled for all patients at baseline and six months after the CXL procedures.

##  Fourier Analysis (corneal shape evaluation)

Fourier analysis transforms corneal keratometric data obtained from the Pentacam device into spherical components (spherical R min, spherical eccentricity), regular astigmatism (central astigmatism, peripheral astigmatism), maximum decentration, and irregularity. The spherical and regular astigmatism components describe standard clinical parameters that can be compensated by spectacles. Decentration is a tilt of the corneal apex with respect to the video-keratoscope axis, and irregularity refers to a range of optical imperfections that degrade retinal image quality.

##  Surgical Technique

In all cases, Glaupin 2%Ⓡ (pilocarpine 2%, Sina Darou, Tehran, Iran) eye drops were applied an hour before the surgery to assure miosis during the procedure and Anestocaine 0.5%Ⓡ (tetracaine 0.5%, Sina Darou, Tehran, Iran) eye drops were applied just before the surgery to provide topical anesthesia. In the epithelium-removal group, 8.5 mm of corneal epithelium was removed completely using a FUKASAKU Hockey Knife (Millennium Surgical Corp, Pennsylvania, USA). A standard preservative solution plus dextran-free riboflavin 0.1% (Sina Darou, Tehran, Iran) was applied at 3-min intervals for 30 min. This step was followed by corneal irradiation with UV-A at a wavelength of 365 nm and power of 9 mW/cm${}^{2}$ at a short distance from the eye (about 5 cm) for 10 min using a CCL-365-vario (MLase AG, Germering, Germany). Riboflavin was instilled every 3 min during corneal irradiation. Finally, the eye was irrigated with 30 ml of balanced salt solution (BSS) and a bandage contact lens (BCL) (CIBA Vision, Duluth, GA, USA) was fitted over the cornea.

In the other eye, the corneal epithelium was disrupted using a DAYA epithelium disruptor (Duckworth & Kent Ltd, Hertfordshire, UK); the rest of the procedure was performed the same way as that described earlier. Ciprofloxacin 0.3% eye drop was instilled every 6 hours for a week and Betamethasone 0.1% eye drop was instilled every 8 hours for a month in the operated eye, postoperatively.

BCL was removed after corneal epithelial defects completely healed. After removing the BCL, patients were visited at weeks 1 and 3 to assess whether there were any complications. Final follow-up examination was performed six months after the second procedure and included slit-lamp biomicroscopy, refraction, UCVA, BSCVA, and PentacamⓇ imaging.

##  Statistical Analysis

Categorical variables are expressed as frequency (percentage) and continuous variables are expressed as the mean value ± standard deviation (SD). Normality of the data was evaluated using the Kolmogorov–Smirnov test. A paired-samples *t*-test or Wilcoxon test was used to compare preoperative and postoperative data. The study groups were compared using Student's *t*-test or the Mann–Whitney U-test. A *P*-value < 0.05 was considered statistically significant.

##  RESULTS 

A total of 68 eyes of 34 patients (10 men and 24 women) with bilateral keratoconus were included in this RCT. The mean age of the participants was 23.3 ± 3.6 years, ranging from 18 to 33 years. Each eye of the patients was randomly assigned to either the epithelium-removal CXL group (*n* = 34) or epithelium-disruption CXL group (*n* = 34). Except five patients who underwent CXL procedure unilaterally, the CXL was performed bilaterally in other subjects. Finally, the epithelium-removal and the epithelium-disruption CXL groups comprised 32 and 31 eyes, respectively. The baseline demographic and clinical characteristics and Fourier transform components were matched between the two groups [Table 1].

**Table 1:** Baseline characteristics of the epithelium removal and epithelial-disruption CXL groups.


**Variables**	**Epithelium-removal CXL (n=32)** Mean ± SD	**Epithelial-disruption CXL (n=31)** Mean ± SD	**** ***P*** **-value**
Sex (m/f)	(31.3% / 68.7%)	(29% / 71%)	0.84
Age (years)	23.40 ± 3.80	23.20 ± 3.50	0.81
Sph equivalent (D)	–2.70 ± 2.70	–3.90 ± 3.20	0.20
BSCVA (logMAR)	0.20 ± 0.20	0.19 ± 0.23	0.50
UCVA (logMAR)	0.55 ± 0.34	0.50 ± 0.37	0.69
Mean keratometry (D)	47.60 ± 3.10	48.20 ± 3.20	0.46
Thinnest point Pachymetry (µm)	459.20 ± 37.40	455.80 ± 32.70	0.70
**Fourier analysis**			
Spherical R min	6.97 ± 0.56	6.8 ±0.64	0.56
Spherical ecc	0.79 ± 0.24	0.87 ± 0.21	0.33
Max decentration	0.78 ± 0.37	0.76 ± 0.35	0.95
Central astigmatism	0.31 ± 0.19	0.27 ± 0.12	0.75
Peripheral astigmatism	0.17 ± 0.11	0.18 ± 0.08	0.81
Irregularity	0.03 ± 0.016	0.028 ± 0.011	0.74
	
	
CXL, corneal crosslinking; BSCVA, best spectacle-corrected visual acuity; CXL, crosslinking; D, diopter; f, female; logMAR, log of the minimal angle of resolution; m, male; Sph, spherical; UCVA, uncorrected visual acuity; µm, micrometer			

**Table 2:** Clinical outcomes of epithelium removal CXL and epithelial-disruption CXL after a mean follow-up of six months


**Variables**	**Epithelium-off CXL **Mean ± SD	**Daya epithelial disruption CXL **Mean ± SD	**** ***P*** **-value**
Spherical equivalent (D)	Before	–2.70 ± 2.70	–3.90 ± 3.20	0.31
	After	–3.00 ± 2.60	–3.70 ± 2.70	
BSCVA (logMAR)	Before	0.20 ± 0.20	0.19 ± 0.23	0.23
	After	0.22 ± 0.22	0.17 ± 0.19	
UCVA (logMAR)	Before	0.55 ± 0.34	0.50 ± 0.37	0.79
	After	0.47 ± 0.34	0.37 ± 0.31	
Mean keratometry (D)	Before	47.60 ± 3.10	48.20 ± 3.20	0.85
	After	47.50 ± 3.30	48.20 ± 3.50	
Thinnest point pachymetry (µm)	Before	459.20 ± 37.4	455.80 ± 32.70	0.0001${}^{*}$
	After	433.50 ± 33.50	451.90 ± 39.70	
**Fourier analysis**				
Spherical R min	Before	6.97 ± 0.56	6.80 ±0.64	0.99
	After	6.90 ± 0.58	6.10 ± 0.66	
Spherical ecc	Before	0.79 ± 0.24	0.87 ± 0.21	0.29
	After	0.84 ± 0.26	0.87 ± 0.21	
Max decentration	Before	0.78 ± 0.37	0.76 ± 0.35	0.92
	After	0.77 ± 0.39	0.72 ± 0.36	
Central astigmatism	Before	0.31 ± 0.19	0.27 ± 0.12	0.85
	After	0.30 ± 0.18	0.28 ± 0.12	
Peripheral astigmatism	Before	0.17 ± 0.11	0.18 ± 0.08	0.49
	After	0.18 ± 0.09	0.18 ± 0.08	
Irregularity	Before	0.030 ± 0.016	0.028 ± 0.011	0.04${}^{*}$
	After	0.031 ± 0.016	0.024 ±0.009	
	
	
BSCVA, best spectacle-corrected visual acuity; CXL, crosslinking; D, diopter; f, female; logMAR, log of the minimal angle of resolution; m, male; UCVA, uncorrected visual acuity; µm, micrometer.				
Indicates for statistically significant *P*-values, (*P* < 0.05)				

**Table 3:** Comparison of epithelium removal CXL with other CXL modifications


**Reference number.**	**Variables**	**Study design**	**Mean k (D)**	**BSCVA (LogMAR)**	**UCVA (LogMAR)**	**CT (µm)**	**F-U (m)**	**Number**
(Li J study)	Epi-removal CXL vs control	Meta-analysis of RCTs	Improvement in CXL Δ = 1.65 *P * < 0.00001	Improvement in CXL Δ = 1.65 *P * < 0.00001	Same *P * > 0.05	Same *P * > 0.05	3–36	Epi-removal = 175 Control = 182
(Li W study)	Epi-removal CXL vs trans-epi CXL	Meta-analysis of RCTs	More improvement in Epi-off CXL Δ = 1.05 *P *= 0.02	More improvement in trans-epi Δ = 0.07 *P *= 0.007	Same *P * > 0.05	Same *P * > 0.05	12–24	Epi-removal = 111 Trans-epi = 133
(Hashemi study)	Epi-removal CXL vs Partial trans-epi CXL (strips pattern)	Retrospective	More improvement in Epi-off Δ = 0.42 *P *= 0.015	More improvement in partial Δ = 0.13 *P *= 0.001	Same *P * > 0.05	Less decrease in partial Δ = 18 *P * < 0.001	12	Epi-removal = 40 Partial = 40
(Current study)	Epi-removal CXL vs epi-disruption	RCT	Same *P * > 0.05	Same *P * > 0.05	Same *P * > 0.05	Less decrease in Epi-disruption Δ = 20 *P *= 0.0001	6	Epi-removal = 32 Partial = 31
	
	
BSCVA, best spectacle-corrected visual acuity; CT, central corneal thickness; CXL, crosslinking; D, diopter; epi, epithelium; F-U, follow-up; logMAR, log of the minimal angle of resolution; m, month; mean k, mean keratometry; RCT, randomized clinical trial; UCVA, uncorrected visual acuity; µm, micrometer; Δ = difference in change between two groups								

Table 2 compares preoperative and postoperative parameters between the two groups. The mean number of days until BCL removal in the conventional and epithelium-disruption CXL groups were 4.5 ± 1.3 and 3.1 ± 1.1 days, respectively (*P* < 0.0001). The two treatment groups differed significantly with respect to the postoperative thickness of the corneal thinnest point (*P *= 0.0001) and the postoperative irregularity component of the Fourier analysis (*P *= 0.04). The epithelium-disruption CXL group had less reduction in the pachymetry of the corneal thinnest point and greater reduction in the Fourier irregularity component in comparison to those in the conventional CXL group. There was no significant difference between the two treatment groups with respect to the other clinical outcomes, such as mean spherical equivalent, keratometry, UCVA, BSCVA, and other components of the Fourier analysis.

##  DISCUSSION

Corneal crosslinking is a procedure that has changed the treatment pathway of keratoconus patients. Before introducing this procedure, a fair prognosis for a young patient diagnosed with keratoconus was expected and the progressive nature of the disease in many cases led to corneal transplantation. However, despite the presence of many patients with advanced keratoconus requiring corneal transplantation, the future is promising for keratoconus treatment.

The major CXL effect is to halt the progression of keratoconus and improve the visual acuity and keratometric data of many patients.^[6,7]^ During the conventional CXL procedure, complete epithelium debridement is performed, which has adverse effects such as pain, increased risk of infection, long healing time, and some stromal haziness.

Patients with advanced keratoconus do not take advantage of conventional treatment methods for visual acuity^[18]^ and pachymetry.^[5]^ Previous studies evaluating the characteristics influencing the outcomes of CXL for keratoconus revealed that patients with a corrected distance visual acuity (CDVA) of 20/40 or worse or a maximum K of 55.0 D or more were most likely to have improvement after CXL.^[18,19]^ Corneal cross-linking in patients with very mild keratoconus and visual acuity of 20/20 is accompanied by the risk of stromal haziness and reduced post-surgical visual acuity.^[15]^ However, in severe cases with low pachymetry (< 400 µm), it might hurt the endothelium and intraocular structures.^[5]^ Epithelial-disruption CXL is a modified version of conventional CXL with reduced adverse effects. In this modified procedure, some controlled pores were induced on the epithelium surface using a DAYA custom-designed instrument to accelerate the diffusion of riboflavin molecules to the stromal layer. In other words, partial removal of the epithelium was implemented. This RCT compared the six month results after epithelium-removal CXL and epithelial-disruption CXL based on the clinical characteristics and Fourier analysis findings.

The results of the current study show that the epithelial-disruption CXL method induces a less reduction in corneal pachymetry in comparison to that induced by conventional CXL after six months. The difference between the mean pachymetry of the thinnest point at baseline and after six-months' follow-up in the epithelial-disruption group was only 5 µm, while it was 25 µm in the epithelium removal CXL group. We suggest this difference is because of the different surgical methods. Epithelial-disruption CXL is a less invasive method in comparison to the conventional CXL and little manipulation of the corneal epithelium is performed through this procedure. This results in a smaller reduction in the mean pachymetry measurement and a corneal thickness closer to the patients' initial corneal thickness six months after the surgery in the epithelial-disruption CXL group. However, previous studies of epithelium removal CXL revealed that after one year of follow-up, postoperative pachymetry reaches its initial measured value.^[20]^ The mean number of days until bandage removal following the epithelium-disruption CXL method was 3.1 days, while it was 4.5 days following the conventional CXL method. After the epithelium-disruption CXL procedure, the corneal ulcer was limited to the many perforations of the epithelial surface, while it was extended due to the completely removed epithelium after the conventional CXL method. Therefore, the number of days before BCL removal was significantly reduced following the epithelium-disruption CXL method. The current results showed faster corneal stabilization following the epithelial-disruption method in comparison to the epithelium removal method. Performing CXL in some advanced cases is risky and may be limited because of a thin cornea. Epithelial-disruption CXL would be a preferable method in this scenario.

There was no difference in the refractive components, UCVA, BSCVA, or mean keratometry results between the two groups. Our findings revealed that although epithelium removal CXL is more aggressive, it does not lead to better short-term follow-up results. We made a comparison between our results and those of previous studies that had compared epithelium removal CXL with other CXL modifications [Table 3]. Hashemi et al compared epithelium removal CXL with partial trans-epithelial CXL (strips pattern de-epithelization) after one year of follow-up.^[13]^ They found better corneal flattening with epithelium removal CXL and less reduction in the central pachymetry results in the partial trans-epithelial CXL group. Their findings of better corneal flattening was not consistent with our results, but their central pachymetry finding was similar to ours.

Our results from the Fourier analysis showed a significant difference in corneal irregularity between the two groups, with better results achieved using the epithelial-disruption CXL method. In other words, patients in the epithelial-disruption group experienced a greater reduction in corneal irregularities than the epithelium removal CXL group after six months. This might be due to the reduced level of manipulation of the epithelium, which reveals the advantage of the epithelial-disruption CXL method over epithelium removal method. Ziaei et al measured anterior corneal curvature variables immediately after epithelial debridement during the CXL procedure.^[21]^ They found that the corneal epithelium plays a role in masking the irregularity of the underlying Bowman's layer in keratoconic eyes. Their results revealed that the magnitude of anterior corneal keratometry, astigmatism, and prolateness significantly increased immediately after epithelial debridement during the CXL procedure, strengthening our results with respect to the preference of the epithelial-disruption CXL method over the epithelium removal CXL method because the former induces less corneal irregularity after the CXL procedure. There was no significant difference in spherical R min, spherical eccentricity, maximum decentration, or central and peripheral astigmatism between the two groups. The results of the Fourier components are compatible with the clinical characteristics findings. There was no difference in either spherical components or spherical equivalent, as well as central astigmatism or mean keratometry. The corneal irregularity component cannot be measured in routine clinical examination, although it is an indicator for distorted image quality after spectacle compensation for refractive error. To the best of our knowledge, no previous studies have compared different CXL methods using Fourier analysis. A comparison between trans-epithelial CXL and epithelium removal CXL in an RCT regarding higher order aberrations via Zernike analysis was performed by Godefrooij et al.^[22]^ They hypothesized that more improvement in BSCVA following trans-epithelial CXL than following epithelium removal CXL was related to greater advancement in the higher order aberrations of the trans-epithelial group relative to the epithelium removal group. Their results did not show any significant difference regarding higher order aberrations between the two groups. The irregularity component of the Fourier analysis might serve as a better answer for that hypothesis.

The study design was a point of strength. It was a double masked RCT where both the patients and the optometrist who collected the clinical data were blinded to the surgical methods. Consequently, a favorable setting to compare treatment effects without confounding factors was provided. In addition, we selected our participants from a pool of patients with bilateral keratoconus and both treatment methods were performed in the same patient. As a result, we examined two groups with similar underlying healing factors. This study addressed the short-term results; consequently, other studies with more than one year of follow-up are recommended to compare the efficacy of these two methods in halting keratoconus progression in the long-term.

In conclusion, the epithelial-disruption CXL method produced better results with respect to the thinnest point on pachymetry and corneal irregularity than the epithelium removal CXL method. There was no significant difference in the improvement in spherical equivalent, mean keratometry, UCVA, BSCVA, or overall shape of the cornea according to Fourier components (except irregularity) between the two CXL methods.

## References

[1] Rabinowitz Y. S. (1998). Keratoconus. *Survey of Ophthalmology*.

[2] Rebenitsch R. L., Kymes S. M., Walline J. J., Gordon M. O. (2011). The lifetime economic burden of keratoconus: a decision analysis using a markov model. *American Journal of Ophthalmology*.

[3] Tatematsu-Ogawa Y., Yamada M., Kawashima M., Yamazaki Y., Bryce T., Tsubota K. (2008). The disease burden of keratoconus in patients' lives: Comparisons to a Japanese normative sample. *Fırat Sağlık Hizmetleri Dergisi*.

[4] Hashemi H., Khabazkhoob M., Fotouhi A. (2013). Topographic keratoconus is not rare in an Iranian population: the Tehran eye study. *Ophthalmic Epidemiology*.

[5] Wollensak G., Spoerl E., Seiler T. (2003). Riboflavin/ultraviolet-a-induced collagen crosslinking for the treatment of keratoconus. *American Journal of Ophthalmology*.

[6] Li J., Ji P., Lin X. L. (2015). Efficacy of corneal collagen cross-linking for treatment of keratoconus: a meta-analysis of randomized controlled trials. *PLoS ONE*.

[7] Bamdad S., Sedaghat M., Bagheri M., Ghavami S. (2015). Changes in corneal topography and biomechanical properties after collagen cross linking for keratoconus: 1-year results. *Middle East African Journal of Ophthalmology*.

[8] Lesniak S. P., Hersh P. S. (2014). Transepithelial corneal collagen crosslinking for keratoconus: six-month results. *Journal of Cataract & Refractive Surgery*.

[9] Li W., Wang B. (2017). Efficacy and safety of transepithelial corneal collagen crosslinking surgery versus standard corneal collagen crosslinking surgery for keratoconus: A meta-analysis of randomized controlled trials. *BMC Ophthalmology*.

[10] Soeters N., Wisse R. P. L., Godefrooij D. A., Imhof S. M., Tahzib N. G. (2015). Transepithelial versus epithelium-off corneal cross-linking for the treatment of progressive keratoconus: a randomized controlled trial. *American Journal of Ophthalmology*.

[11] Rechichi M., Daya S., Scorcia V., Meduri A., Scorcia G. (2013). Epithelial-disruption collagen crosslinking for keratoconus: One-year results. *Journal of Cataract & Refractive Surgery*.

[12] Hirji N., Sykakis E., Lam F. C., Petrarca R., Hamada S., Lake D. (2015). Corneal collagen crosslinking for keratoconus or corneal ectasia without epithelial debridement. *Eye*.

[13] Hashemi H., Miraftab M., Hafezi F., Asgari S. (2015). Matched Comparison Study of Total and Partial Epithelium Removal in Corneal Cross-linking. *Journal of Refractive Surgery*.

[14] Galvis V., Tello A., Carreño N. I., Ortiz A. I., Barrera R., Rodriguez C. J., Ochoa M. E. (2016). Corneal Cross-Linking (with a Partial Deepithelization) in Keratoconus with Five Years of Follow-Up. *Ophthalmology and Eye Diseases*.

[15] Sideroudi H., Labiris G., Ditzel F., Tsaragli E., Georgatzoglou K., Siganos H., Kozobolis V. (2018). Validation of Fourier analysis of videokeratographic data. *International Ophthalmology*.

[16] Sideroudi H., Labiris G., Georgatzoglou K., Ditzel F., Siganos C., Kozobolis V. (2016). Fourier analysis of videokeratography data: Clinical usefulness in grade I and subclinical keratoconus. *Journal of Cataract & Refractive Surgery*.

[17] Oshika T., Tanabe T., Tomidokoro A., Amano S. (2002). Progression of keratoconus assessed by fourier analysis of videokeratography data. *Ophthalmology*.

[18] Greenstein S. A., Hersh P. S. (2013). Characteristics influencing outcomes of corneal collagen crosslinking for keratoconus and ectasia: implications for patient selection. *Journal of Cataract & Refractive Surgery*.

[19] Sloot F., Soeters N., Van Der Valk R., Tahzib N. G. (2013). Effective corneal collagen crosslinking in advanced cases of progressive keratoconus. *Journal of Cataract & Refractive Surgery*.

[20] Greenstein S. A., Shah V. P., Fry K. L., Hersh P. S. (2011). Corneal thickness changes after corneal collagen crosslinking for keratoconus and corneal ectasia: one-year results. *Journal of Cataract & Refractive Surgery*.

[21] Ziaei M., Meyer J., Gokul A., Vellara H., McGhee C. N. (2018). Direct measurement of anterior corneal curvature changes attributable to epithelial removal in keratoconus. *Journal of Cataract & Refractive Surgery*.

[22] Godefrooij D., El Kandoussi M., Soeters N., Wisse R. (2017). Higher order optical aberrations and visual acuity in a randomized controlled trial comparing transepithelial versus epithelium-off corneal crosslinking for progressive keratoconus. *Clinical Ophthalmology*.

